# Bis[bis­(1*H*-pyrazol-1-yl)methane-κ^2^ *N* ^2^,*N* ^2′^](formato-κ^2^ *O*,*O*′)copper(II) perchlorate

**DOI:** 10.1107/S1600536811039675

**Published:** 2011-10-05

**Authors:** Cai-Juan Zhao, Rui-Feng Zhang

**Affiliations:** a School of Chemistry & Material Science, Shanxi Normal University, Linfen 041004, People’s Republic of China

## Abstract

In the crystal structure of the title compound, [Cu(HCO_2_)(C_7_H_8_N_4_)_2_]ClO_4_, the Cu^II^ ion is octa­hedrally coordinated by one bidentate formate ion and two bidentate bis­(1*H*-pyrazol-1-yl)methane ligands. There are C—H⋯O hydrogen bonds and π–π inter­actions [centroid–centroid distance = 3.487  (3) Å] in the crystal structure. The perchlorate anion is disordered over two positions with an occupancy ratio of 0.628 (9):0.372 (9).

## Related literature

For applications of coordination polymers, see: Kitagawa *et al.* (2004 ▶); Robson (2000 ▶). For synthesis of the bis­(pyrazol-1-yl)methane ligand, see: Elguero *et al.* (1982 ▶). 
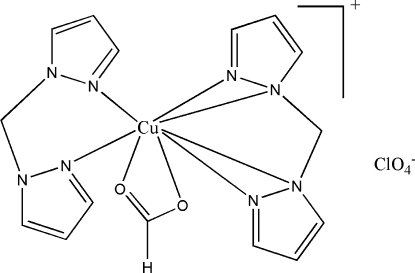


## Experimental

### 

#### Crystal data

[Cu(CHO_2_)(C_7_H_8_N_4_)_2_]ClO_4_
*M* *_r_* = 504.36
Monoclinic, 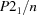

*a* = 11.0458 (19) Å
*b* = 14.816 (3) Å
*c* = 12.273 (2) Å
β = 99.031 (3)°
*V* = 1983.6 (6) Å^3^
*Z* = 4
Mo *K*α radiation
μ = 1.29 mm^−1^
*T* = 294 K
0.22 × 0.20 × 0.16 mm

#### Data collection

Bruker SMART CCD area-detector diffractometer
Absorption correction: multi-scan (*SADABS*; Sheldrick, 1996 ▶) *T*_min_ = 0.768, *T*_max_ = 1.000
9920 measured reflections
3439 independent reflections
2334 reflections with *I* > 2σ(*I*)
*R*_int_ = 0.037

#### Refinement

*R*[*F* ^2^ > 2σ(*F* ^2^)] = 0.052
*wR*(*F* ^2^) = 0.165
*S* = 1.02
3439 reflections
318 parameters
122 restraints
H-atom parameters constrained
Δρ_max_ = 1.07 e Å^−3^
Δρ_min_ = −0.62 e Å^−3^

### 

Data collection: *SMART-NT* (Bruker, 1998 ▶); cell refinement: *SAINT-NT* (Bruker, 1998 ▶); data reduction: *SAINT-NT*; program(s) used to solve structure: *SHELXS97* (Sheldrick, 2008 ▶); program(s) used to refine structure: *SHELXL97* (Sheldrick, 2008 ▶); molecular graphics: *XP* in *SHELXTL* (Sheldrick, 2008 ▶); software used to prepare material for publication: *SHELXTL*.

## Supplementary Material

Crystal structure: contains datablock(s) global, I. DOI: 10.1107/S1600536811039675/jh2326sup1.cif

Structure factors: contains datablock(s) I. DOI: 10.1107/S1600536811039675/jh2326Isup2.hkl

Additional supplementary materials:  crystallographic information; 3D view; checkCIF report

## Figures and Tables

**Table 1:** Hydrogen-bond geometry (Å, °)

*D*—H⋯*A*	*D*—H	H⋯*A*	*D*⋯*A*	*D*—H⋯*A*
C13—H13⋯O2^i^	0.93	2.66	3.424 (7)	140
C4—H4*B*⋯O4^ii^	0.97	2.37	3.343 (14)	176
C4—H4*B*⋯O4′^ii^	0.97	2.66	3.580 (12)	159
C11—H11*B*⋯O5^iii^	0.97	2.60	3.525 (17)	161
C11—H11*B*⋯O5′^iii^	0.97	2.32	3.286 (9)	176
C12—H12⋯O3^iii^	0.93	2.49	3.220 (14)	136
C12—H12⋯O3′^iii^	0.93	2.30	3.195 (10)	161

Symmetry codes: (i) 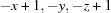; (ii) 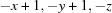; (iii) 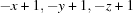.

**Fig. 1.**
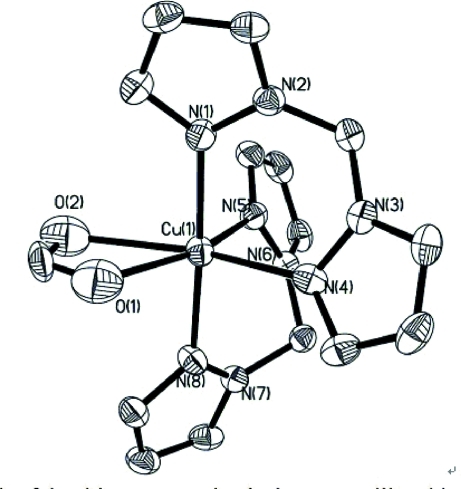
The asymmetric unit of the title compound. Displacement ellipsoids are drawn at the 30% probability level. (Hydrogen atoms and the perchlorate ion are omitted for clarity.)

**Fig. 2.**
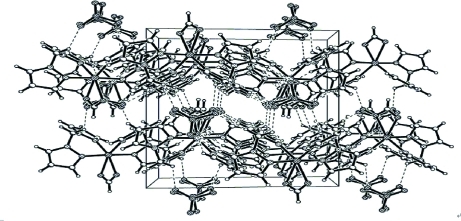
The packing diagram of the title compound. Hydrogen bonds are shown as dashed lines.

## supplementary crystallographic information

### Comment

Coordination polymers have received significant attention in recent years, primarily due to their potential applications in many areas such as catalysis, molecular adsorption, magnetism properties and non-linear optics (Kitagawa *et al.*, 2004; Robson, 2000). We report herein the structure of the title compound, namely [Cu(*L*_1_)_2_(HCO_2_)]ClO_4_ (*L*_1_ = bis(pyrazol-1-yl)methane). The title compound crystallizes in monoclinic space group P2_1_/n. The asymmetrical unit of the unit cell contains one Cu^II^ ion, one formic acid and two ligand *L*_1_ (as shown in Fig. 1). The Cu ion is octahedrally coordinated to two oxygen atoms from one formate ion and four nitrogen atoms from two *L*_1_ ligands. In the crystal structure, intermolecular C—H···O hydrogen bonds link the molecules into a three-dimensional network (Fig. 2), and π-π interactions between two pyrazole rings (centroid-centroid distance is 3.487 Å) consolidate the crystal packing.

### Experimental

The ligand *L*_1_ was synthesized according to literature (Elguero *et al.*, 1982). The title compound was prepared by adding 5 ml methanol solution of copper(II) perchlorate (0.3 mmol) to 10 ml aqueous solution of *L*_1_ (0.5 mmol) and formic acid (0.3 mmol). The mixture was stirred for half an hour and filtered. The filtrate was slowly evaporated at room temperature to yield blue cubic crystals suitable for X-ray analysis. Analysis calculated for C_15_H_17_ClCuN_8_: C 35.69, H 3.37, N 22.21%; found: C 33.21, H 3.09, N 24.03%.

### Refinement

Hydrogen atoms were included in calculated positions and refined with fixed thermal parameters riding on their parent atoms with C—H distances in the range of 0.93–0.97 Å, *U*_iso_(H) = 1.2*U*_eq_(C).

### Crystal data

**Table:**

[Cu(CHO_2_)(C_7_H_8_N_4_)_2_]ClO_4_	*F*(000) = 1028
*M**_r_* = 504.36	*D*_x_ = 1.689 Mg m^−^^3^
Monoclinic, *P*2_1_/*n*	Mo *K*α radiation, λ = 0.71073 Å
*a* = 11.0458 (19) Å	Cell parameters from 3002 reflections
*b* = 14.816 (3) Å	θ = 2.2–25.4°
*c* = 12.273 (2) Å	µ = 1.29 mm^−^^1^
β = 99.031 (3)°	*T* = 294 K
*V* = 1983.6 (6) Å^3^	Cubic, blue
*Z* = 4	0.22 × 0.20 × 0.16 mm

### Data collection

**Table:**

Bruker SMART CCD area-detector diffractometer	3439 independent reflections
Radiation source: fine-focus sealed tube	2334 reflections with *I* > 2σ(*I*)
graphite	*R*_int_ = 0.037
φ and ω scans	θ_max_ = 25.0°, θ_min_ = 2.2°
Absorption correction: multi-scan (*SADABS*; Sheldrick, 1996)	*h* = −8→13
*T*_min_ = 0.768, *T*_max_ = 1.000	*k* = −17→16
9920 measured reflections	*l* = −14→14

### Refinement

**Table:**

Refinement on *F*^2^	Secondary atom site location: difference Fourier map
Least-squares matrix: full	Hydrogen site location: inferred from neighbouring sites
*R*[*F*^2^ > 2σ(*F*^2^)] = 0.052	H-atom parameters constrained
*wR*(*F*^2^) = 0.165	*w* = 1/[σ^2^(*F*_o_^2^) + (0.099*P*)^2^ + 1.4485*P*] where *P* = (*F*_o_^2^ + 2*F*_c_^2^)/3
*S* = 1.02	(Δ/σ)_max_ = 0.001
3439 reflections	Δρ_max_ = 1.07 e Å^−^^3^
318 parameters	Δρ_min_ = −0.62 e Å^−^^3^
122 restraints	Extinction correction: *SHELXL97* (Sheldrick, 2008), Fc^*^=kFc[1+0.001xFc^2^λ^3^/sin(2θ)]^-1/4^
Primary atom site location: structure-invariant direct methods	Extinction coefficient: 0.0099 (12)

### Special details

**Table:**

Geometry. All e.s.d.'s (except the e.s.d. in the dihedral angle between two l.s. planes) are estimated using the full covariance matrix. The cell e.s.d.'s are taken into account individually in the estimation of e.s.d.'s in distances, angles and torsion angles; correlations between e.s.d.'s in cell parameters are only used when they are defined by crystal symmetry. An approximate (isotropic) treatment of cell e.s.d.'s is used for estimating e.s.d.'s involving l.s. planes.
Refinement. Refinement of *F*^2^ against ALL reflections. The weighted *R*-factor *wR* and goodness of fit *S* are based on *F*^2^, conventional *R*-factors *R* are based on *F*, with *F* set to zero for negative *F*^2^. The threshold expression of *F*^2^ > σ(*F*^2^) is used only for calculating *R*-factors(gt) *etc*. and is not relevant to the choice of reflections for refinement. *R*-factors based on *F*^2^ are statistically about twice as large as those based on *F*, and *R*- factors based on ALL data will be even larger.

### Fractional atomic coordinates and isotropic or equivalent isotropic displacement parameters (Å^2^)

**Table:**

	*x*	*y*	*z*	*U*_iso_*/*U*_eq_	Occ. (<1)
Cu1	0.51417 (5)	0.18886 (4)	0.24183 (4)	0.0406 (3)	
O1	0.4358 (5)	0.0590 (4)	0.1916 (4)	0.0968 (16)	
O2	0.5977 (5)	0.0465 (4)	0.2919 (4)	0.0984 (16)	
N1	0.6033 (4)	0.1867 (3)	0.1089 (3)	0.0448 (9)	
N2	0.5865 (3)	0.2511 (3)	0.0313 (3)	0.0431 (9)	
N3	0.4141 (3)	0.3339 (2)	0.0709 (3)	0.0410 (9)	
N4	0.3848 (3)	0.2847 (3)	0.1554 (3)	0.0451 (10)	
N5	0.6403 (3)	0.2826 (3)	0.3261 (3)	0.0427 (9)	
N6	0.6134 (3)	0.3316 (2)	0.4117 (3)	0.0388 (9)	
N7	0.4428 (3)	0.2470 (2)	0.4540 (3)	0.0405 (9)	
N8	0.4262 (4)	0.1837 (2)	0.3739 (3)	0.0457 (9)	
C1	0.6507 (5)	0.1161 (3)	0.0628 (4)	0.0550 (13)	
H1	0.6716	0.0613	0.0978	0.066*	
C2	0.6644 (5)	0.1366 (4)	−0.0450 (4)	0.0559 (13)	
H2	0.6959	0.0993	−0.0946	0.067*	
C3	0.6224 (4)	0.2217 (4)	−0.0628 (4)	0.0504 (12)	
H3	0.6188	0.2543	−0.1281	0.060*	
C4	0.5405 (4)	0.3388 (3)	0.0552 (4)	0.0435 (11)	
H4A	0.5896	0.3628	0.1213	0.052*	
H4B	0.5478	0.3796	−0.0053	0.052*	
C5	0.3162 (5)	0.3769 (3)	0.0150 (4)	0.0504 (12)	
H5	0.3156	0.4142	−0.0460	0.061*	
C6	0.2190 (5)	0.3555 (4)	0.0647 (5)	0.0593 (14)	
H6	0.1385	0.3750	0.0451	0.071*	
C7	0.2642 (4)	0.2986 (4)	0.1508 (4)	0.0532 (13)	
H7	0.2170	0.2732	0.1993	0.064*	
C8	0.7606 (5)	0.2959 (3)	0.3287 (4)	0.0518 (13)	
H8	0.8065	0.2702	0.2793	0.062*	
C9	0.8077 (5)	0.3525 (4)	0.4143 (5)	0.0570 (13)	
H9	0.8884	0.3718	0.4326	0.068*	
C10	0.7118 (4)	0.3739 (3)	0.4658 (4)	0.0496 (12)	
H10	0.7140	0.4109	0.5272	0.059*	
C11	0.4874 (4)	0.3361 (3)	0.4292 (4)	0.0417 (10)	
H11A	0.4371	0.3598	0.3635	0.050*	
H11B	0.4808	0.3767	0.4899	0.050*	
C12	0.4066 (4)	0.2174 (4)	0.5466 (4)	0.0522 (13)	
H12	0.4091	0.2498	0.6118	0.063*	
C13	0.3656 (5)	0.1318 (4)	0.5284 (4)	0.0574 (14)	
H13	0.3350	0.0940	0.5781	0.069*	
C14	0.3787 (5)	0.1122 (3)	0.4204 (4)	0.0513 (12)	
H14	0.3577	0.0576	0.3851	0.062*	
C15	0.5126 (7)	0.0079 (4)	0.2389 (5)	0.0663 (18)	
H15	0.5063	−0.0547	0.2345	0.080*	
Cl1	0.48695 (14)	0.56011 (9)	0.25933 (10)	0.0592 (4)	
O3	0.4395 (14)	0.6517 (6)	0.2731 (10)	0.094 (5)	0.372 (9)
O4	0.4250 (15)	0.5272 (9)	0.1570 (8)	0.110 (6)	0.372 (9)
O5	0.4583 (17)	0.5091 (9)	0.3505 (10)	0.145 (7)	0.372 (9)
O6	0.6157 (8)	0.5647 (14)	0.2589 (18)	0.267 (14)	0.372 (9)
O3'	0.5275 (10)	0.6523 (5)	0.2451 (7)	0.125 (4)	0.628 (9)
O4'	0.5199 (12)	0.5081 (7)	0.1726 (8)	0.147 (4)	0.628 (9)
O5'	0.5488 (9)	0.5305 (5)	0.3645 (5)	0.094 (3)	0.628 (9)
O6'	0.3574 (7)	0.5562 (10)	0.2584 (13)	0.259 (9)	0.628 (9)

### Atomic displacement parameters (Å^2^)

**Table:**

	*U*^11^	*U*^22^	*U*^33^	*U*^12^	*U*^13^	*U*^23^
Cu1	0.0503 (4)	0.0379 (4)	0.0332 (4)	−0.0003 (2)	0.0051 (2)	0.0002 (2)
O1	0.109 (4)	0.118 (4)	0.067 (3)	−0.004 (3)	0.023 (3)	−0.008 (3)
O2	0.108 (4)	0.127 (5)	0.063 (3)	−0.003 (3)	0.021 (3)	0.007 (3)
N1	0.053 (2)	0.044 (2)	0.037 (2)	0.0068 (18)	0.0069 (18)	0.0010 (18)
N2	0.044 (2)	0.048 (2)	0.038 (2)	0.0015 (17)	0.0081 (17)	0.0010 (18)
N3	0.042 (2)	0.039 (2)	0.040 (2)	0.0013 (16)	0.0004 (17)	−0.0036 (17)
N4	0.044 (2)	0.055 (2)	0.036 (2)	−0.0080 (18)	0.0048 (18)	0.0002 (18)
N5	0.044 (2)	0.046 (2)	0.038 (2)	0.0073 (17)	0.0067 (17)	0.0045 (17)
N6	0.041 (2)	0.039 (2)	0.0358 (19)	−0.0028 (16)	0.0023 (17)	−0.0004 (16)
N7	0.041 (2)	0.042 (2)	0.040 (2)	−0.0030 (16)	0.0080 (17)	−0.0003 (17)
N8	0.052 (2)	0.041 (2)	0.044 (2)	−0.0103 (18)	0.0100 (18)	0.0011 (18)
C1	0.057 (3)	0.050 (3)	0.060 (3)	0.008 (2)	0.014 (3)	−0.005 (2)
C2	0.052 (3)	0.066 (4)	0.052 (3)	−0.004 (3)	0.016 (2)	−0.016 (3)
C3	0.051 (3)	0.064 (3)	0.038 (2)	−0.002 (2)	0.011 (2)	−0.002 (2)
C4	0.047 (3)	0.040 (3)	0.043 (2)	−0.001 (2)	0.004 (2)	0.005 (2)
C5	0.056 (3)	0.042 (3)	0.049 (3)	0.006 (2)	−0.006 (2)	−0.003 (2)
C6	0.040 (3)	0.057 (3)	0.075 (4)	0.011 (2)	−0.008 (3)	−0.016 (3)
C7	0.041 (3)	0.064 (3)	0.056 (3)	−0.003 (2)	0.009 (2)	−0.015 (3)
C8	0.048 (3)	0.055 (3)	0.055 (3)	0.005 (2)	0.014 (2)	0.016 (2)
C9	0.043 (3)	0.057 (3)	0.068 (3)	−0.009 (2)	−0.003 (3)	0.018 (3)
C10	0.051 (3)	0.039 (3)	0.055 (3)	−0.008 (2)	−0.005 (2)	0.003 (2)
C11	0.042 (2)	0.040 (3)	0.043 (2)	0.0008 (19)	0.007 (2)	−0.004 (2)
C12	0.045 (3)	0.074 (4)	0.039 (3)	0.001 (2)	0.012 (2)	0.004 (2)
C13	0.054 (3)	0.070 (4)	0.052 (3)	0.002 (3)	0.017 (3)	0.018 (3)
C14	0.056 (3)	0.043 (3)	0.057 (3)	−0.007 (2)	0.015 (2)	0.002 (2)
C15	0.111 (6)	0.027 (3)	0.067 (4)	−0.004 (3)	0.032 (4)	−0.001 (3)
Cl1	0.0867 (10)	0.0433 (8)	0.0434 (7)	0.0043 (6)	−0.0028 (7)	−0.0003 (5)
O3	0.114 (9)	0.069 (7)	0.095 (8)	0.029 (6)	0.004 (7)	−0.013 (6)
O4	0.136 (10)	0.099 (8)	0.087 (8)	−0.010 (7)	−0.006 (7)	−0.005 (7)
O5	0.145 (7)	0.145 (7)	0.144 (7)	−0.0001 (11)	0.0230 (16)	0.0005 (11)
O6	0.267 (14)	0.267 (14)	0.267 (14)	0.0001 (11)	0.042 (2)	0.0000 (11)
O3'	0.156 (8)	0.089 (6)	0.121 (7)	−0.025 (5)	−0.004 (6)	0.010 (5)
O4'	0.167 (8)	0.148 (8)	0.137 (7)	−0.020 (6)	0.057 (6)	−0.036 (6)
O5'	0.130 (6)	0.074 (5)	0.070 (4)	−0.004 (4)	−0.011 (4)	0.026 (4)
O6'	0.216 (11)	0.289 (13)	0.272 (13)	0.000 (9)	0.038 (9)	−0.026 (9)

### Geometric parameters (Å, °)

**Table:**

Cu1—N8	2.018 (4)	C4—H4A	0.9700
Cu1—N1	2.034 (4)	C4—H4B	0.9700
Cu1—N5	2.119 (4)	C5—C6	1.353 (7)
Cu1—O1	2.160 (5)	C5—H5	0.9300
Cu1—N4	2.169 (4)	C6—C7	1.383 (8)
Cu1—O2	2.345 (6)	C6—H6	0.9300
O1—C15	1.215 (7)	C7—H7	0.9300
O2—C15	1.201 (7)	C8—C9	1.380 (8)
N1—C1	1.334 (6)	C8—H8	0.9300
N1—N2	1.340 (5)	C9—C10	1.354 (8)
N2—C3	1.350 (6)	C9—H9	0.9300
N2—C4	1.443 (6)	C10—H10	0.9300
N3—C5	1.346 (6)	C11—H11A	0.9700
N3—N4	1.347 (5)	C11—H11B	0.9700
N3—C4	1.441 (6)	C12—C13	1.353 (8)
N4—C7	1.340 (6)	C12—H12	0.9300
N5—C8	1.339 (6)	C13—C14	1.388 (7)
N5—N6	1.347 (5)	C13—H13	0.9300
N6—C10	1.338 (6)	C14—H14	0.9300
N6—C11	1.442 (6)	C15—H15	0.9300
N7—C12	1.338 (6)	Cl1—O4'	1.408 (6)
N7—N8	1.350 (5)	Cl1—O4	1.419 (7)
N7—C11	1.458 (6)	Cl1—O6	1.425 (8)
N8—C14	1.347 (6)	Cl1—O5	1.427 (8)
C1—C2	1.388 (7)	Cl1—O6'	1.431 (7)
C1—H1	0.9300	Cl1—O5'	1.432 (5)
C2—C3	1.351 (7)	Cl1—O3'	1.456 (6)
C2—H2	0.9300	Cl1—O3	1.473 (7)
C3—H3	0.9300		
			
N8—Cu1—N1	176.90 (16)	C5—C6—H6	127.2
N8—Cu1—N5	89.75 (15)	C7—C6—H6	127.2
N1—Cu1—N5	92.19 (15)	N4—C7—C6	111.3 (5)
N8—Cu1—O1	88.46 (16)	N4—C7—H7	124.4
N1—Cu1—O1	88.83 (17)	C6—C7—H7	124.4
N5—Cu1—O1	157.97 (19)	N5—C8—C9	111.2 (5)
N8—Cu1—N4	93.12 (15)	N5—C8—H8	124.4
N1—Cu1—N4	89.00 (15)	C9—C8—H8	124.4
N5—Cu1—N4	98.13 (16)	C10—C9—C8	105.6 (4)
O1—Cu1—N4	103.89 (19)	C10—C9—H9	127.2
N8—Cu1—O2	88.52 (16)	C8—C9—H9	127.2
N1—Cu1—O2	88.64 (16)	N6—C10—C9	107.0 (5)
N5—Cu1—O2	105.09 (18)	N6—C10—H10	126.5
O1—Cu1—O2	52.9 (2)	C9—C10—H10	126.5
N4—Cu1—O2	156.73 (18)	N6—C11—N7	110.8 (3)
C15—O1—Cu1	101.5 (4)	N6—C11—H11A	109.5
C15—O2—Cu1	92.5 (4)	N7—C11—H11A	109.5
C1—N1—N2	106.0 (4)	N6—C11—H11B	109.5
C1—N1—Cu1	128.6 (3)	N7—C11—H11B	109.5
N2—N1—Cu1	121.9 (3)	H11A—C11—H11B	108.1
N1—N2—C3	110.7 (4)	N7—C12—C13	107.7 (4)
N1—N2—C4	120.7 (4)	N7—C12—H12	126.2
C3—N2—C4	128.6 (4)	C13—C12—H12	126.2
C5—N3—N4	112.2 (4)	C12—C13—C14	105.7 (4)
C5—N3—C4	128.7 (4)	C12—C13—H13	127.1
N4—N3—C4	119.0 (4)	C14—C13—H13	127.1
C7—N4—N3	104.0 (4)	N8—C14—C13	110.3 (5)
C7—N4—Cu1	134.0 (3)	N8—C14—H14	124.9
N3—N4—Cu1	120.7 (3)	C13—C14—H14	124.9
C8—N5—N6	104.0 (4)	O2—C15—O1	113.0 (6)
C8—N5—Cu1	133.0 (3)	O2—C15—H15	123.5
N6—N5—Cu1	121.7 (3)	O1—C15—H15	123.5
C10—N6—N5	112.2 (4)	O4'—Cl1—O4	44.7 (6)
C10—N6—C11	129.2 (4)	O4'—Cl1—O6	69.3 (8)
N5—N6—C11	118.5 (4)	O4—Cl1—O6	110.9 (7)
C12—N7—N8	111.5 (4)	O4'—Cl1—O5	114.8 (9)
C12—N7—C11	129.0 (4)	O4—Cl1—O5	112.1 (7)
N8—N7—C11	119.3 (3)	O6—Cl1—O5	111.9 (7)
C14—N8—N7	104.8 (4)	O4'—Cl1—O6'	110.4 (6)
C14—N8—Cu1	129.7 (3)	O4—Cl1—O6'	68.7 (7)
N7—N8—Cu1	122.0 (3)	O6—Cl1—O6'	179.2 (9)
N1—C1—C2	109.9 (5)	O5—Cl1—O6'	68.9 (8)
N1—C1—H1	125.0	O4'—Cl1—O5'	111.8 (6)
C2—C1—H1	125.0	O4—Cl1—O5'	142.0 (7)
C3—C2—C1	105.8 (4)	O6—Cl1—O5'	71.5 (8)
C3—C2—H2	127.1	O5—Cl1—O5'	42.6 (7)
C1—C2—H2	127.1	O6'—Cl1—O5'	109.3 (6)
N2—C3—C2	107.5 (4)	O4'—Cl1—O3'	107.6 (5)
N2—C3—H3	126.2	O4—Cl1—O3'	109.3 (7)
C2—C3—H3	126.2	O6—Cl1—O3'	68.2 (9)
N3—C4—N2	111.2 (4)	O5—Cl1—O3'	134.5 (8)
N3—C4—H4A	109.4	O6'—Cl1—O3'	111.3 (6)
N2—C4—H4A	109.4	O5'—Cl1—O3'	106.4 (4)
N3—C4—H4B	109.4	O4'—Cl1—O3	136.9 (7)
N2—C4—H4B	109.4	O4—Cl1—O3	106.7 (6)
H4A—C4—H4B	108.0	O6—Cl1—O3	109.2 (7)
N3—C5—C6	106.9 (5)	O5—Cl1—O3	105.7 (6)
N3—C5—H5	126.6	O6'—Cl1—O3	70.4 (8)
C6—C5—H5	126.6	O5'—Cl1—O3	107.8 (6)
C5—C6—C7	105.7 (4)	O3'—Cl1—O3	43.3 (7)
			
N8—Cu1—O1—C15	−89.2 (4)	C8—N5—N6—C11	176.7 (4)
N1—Cu1—O1—C15	89.3 (4)	Cu1—N5—N6—C11	−14.9 (5)
N5—Cu1—O1—C15	−3.7 (7)	C12—N7—N8—C14	−0.6 (5)
N4—Cu1—O1—C15	178.0 (4)	C11—N7—N8—C14	−176.6 (4)
O2—Cu1—O1—C15	0.1 (3)	C12—N7—N8—Cu1	−161.4 (3)
N8—Cu1—O2—C15	89.1 (4)	C11—N7—N8—Cu1	22.6 (5)
N1—Cu1—O2—C15	−89.7 (4)	N1—Cu1—N8—C14	−10 (3)
N5—Cu1—O2—C15	178.4 (3)	N5—Cu1—N8—C14	−139.0 (5)
O1—Cu1—O2—C15	−0.1 (3)	O1—Cu1—N8—C14	19.0 (5)
N4—Cu1—O2—C15	−5.4 (6)	N4—Cu1—N8—C14	122.8 (4)
N8—Cu1—N1—C1	−6(3)	O2—Cu1—N8—C14	−33.9 (5)
N5—Cu1—N1—C1	123.0 (4)	N1—Cu1—N8—N7	145 (3)
O1—Cu1—N1—C1	−35.0 (5)	N5—Cu1—N8—N7	16.5 (4)
N4—Cu1—N1—C1	−138.9 (4)	O1—Cu1—N8—N7	174.6 (4)
O2—Cu1—N1—C1	17.9 (5)	N4—Cu1—N8—N7	−81.6 (4)
N8—Cu1—N1—N2	150 (3)	O2—Cu1—N8—N7	121.6 (4)
N5—Cu1—N1—N2	−81.0 (3)	N2—N1—C1—C2	0.2 (6)
O1—Cu1—N1—N2	121.0 (4)	Cu1—N1—C1—C2	159.2 (4)
N4—Cu1—N1—N2	17.1 (3)	N1—C1—C2—C3	−0.6 (6)
O2—Cu1—N1—N2	174.0 (4)	N1—N2—C3—C2	−0.6 (5)
C1—N1—N2—C3	0.2 (5)	C4—N2—C3—C2	176.9 (4)
Cu1—N1—N2—C3	−160.5 (3)	C1—C2—C3—N2	0.7 (6)
C1—N1—N2—C4	−177.5 (4)	C5—N3—C4—N2	−121.3 (5)
Cu1—N1—N2—C4	21.8 (5)	N4—N3—C4—N2	62.9 (5)
C5—N3—N4—C7	−0.1 (5)	N1—N2—C4—N3	−69.0 (5)
C4—N3—N4—C7	176.3 (4)	C3—N2—C4—N3	113.8 (5)
C5—N3—N4—Cu1	168.7 (3)	N4—N3—C5—C6	0.2 (5)
C4—N3—N4—Cu1	−14.9 (5)	C4—N3—C5—C6	−175.9 (4)
N8—Cu1—N4—C7	−33.5 (4)	N3—C5—C6—C7	−0.1 (6)
N1—Cu1—N4—C7	144.3 (4)	N3—N4—C7—C6	0.0 (5)
N5—Cu1—N4—C7	−123.7 (4)	Cu1—N4—C7—C6	−166.6 (4)
O1—Cu1—N4—C7	55.7 (5)	C5—C6—C7—N4	0.1 (6)
O2—Cu1—N4—C7	60.1 (6)	N6—N5—C8—C9	−0.3 (5)
N8—Cu1—N4—N3	161.7 (3)	Cu1—N5—C8—C9	−166.8 (3)
N1—Cu1—N4—N3	−20.6 (3)	N5—C8—C9—C10	0.4 (6)
N5—Cu1—N4—N3	71.5 (3)	N5—N6—C10—C9	0.1 (5)
O1—Cu1—N4—N3	−109.1 (3)	C11—N6—C10—C9	−176.0 (4)
O2—Cu1—N4—N3	−104.8 (4)	C8—C9—C10—N6	−0.3 (5)
N8—Cu1—N5—C8	143.9 (4)	C10—N6—C11—N7	−120.8 (5)
N1—Cu1—N5—C8	−33.7 (4)	N5—N6—C11—N7	63.2 (5)
O1—Cu1—N5—C8	58.6 (6)	C12—N7—C11—N6	115.5 (5)
N4—Cu1—N5—C8	−123.0 (4)	N8—N7—C11—N6	−69.2 (5)
O2—Cu1—N5—C8	55.5 (4)	N8—N7—C12—C13	0.5 (6)
N8—Cu1—N5—N6	−20.7 (3)	C11—N7—C12—C13	176.1 (4)
N1—Cu1—N5—N6	161.7 (3)	N7—C12—C13—C14	−0.3 (6)
O1—Cu1—N5—N6	−106.0 (5)	N7—N8—C14—C13	0.4 (6)
N4—Cu1—N5—N6	72.4 (3)	Cu1—N8—C14—C13	159.1 (4)
O2—Cu1—N5—N6	−109.1 (3)	C12—C13—C14—N8	0.0 (6)
C8—N5—N6—C10	0.1 (5)	Cu1—O2—C15—O1	0.2 (5)
Cu1—N5—N6—C10	168.5 (3)	Cu1—O1—C15—O2	−0.2 (6)

### Hydrogen-bond geometry (Å, °)

**Table:**

*D*—H···*A*	*D*—H	H···*A*	*D*···*A*	*D*—H···*A*
C13—H13···O2^i^	0.93	2.66	3.424 (7)	140.
C4—H4B···O4^ii^	0.97	2.37	3.343 (14)	176.
C4—H4B···O4'^ii^	0.97	2.66	3.580 (12)	159.
C11—H11B···O5^iii^	0.97	2.60	3.525 (17)	161.
C11—H11B···O5'^iii^	0.97	2.32	3.286 (9)	176.
C12—H12···O3^iii^	0.93	2.49	3.220 (14)	136.
C12—H12···O3'^iii^	0.93	2.30	3.195 (10)	161.

Symmetry codes: (i) −*x*+1, −*y*, −*z*+1; (ii) −*x*+1, −*y*+1, −*z*; (iii) −*x*+1, −*y*+1, −*z*+1.
